# Development of a validated assessment tool for medical students using simulated patients: an 8-year panel survey

**DOI:** 10.1186/s12909-024-05386-2

**Published:** 2024-04-10

**Authors:** Junji Haruta, Rika Nakajima, Toshiaki Monkawa

**Affiliations:** Medical Education Center, School of Medicine, Keio University, 35 Shinanomachi, Shinjuku-ku, 160-8582 Tokyo, Japan

**Keywords:** Simulated patient, Assessment tools, Validation, Standardization

## Abstract

**Background:**

The use of simulated patients (SPs) to assess medical students’ clinical performance is gaining prominence, underscored by patient safety perspective. However, few reports have investigated the validity of such assessment. Here, we examined the validity and reliability of an assessment tool that serves as a standardized tool for SPs to assess medical students’ medical interview.

**Methods:**

This longitudinal survey was conducted at Keio University School of Medicine in Japan from 2014 to 2021. To establish content validity, the simulated patient assessment tool (SPAT) was developed by several medical education specialists from 2008 to 2013. A cohort of 36 SPs assessed the performance of 831 medical students in clinical practice medical interview sessions from April 2014 to December 2021. The assessment’s internal structure was analyzed using descriptive statistics (maximum, minimum, median, mean, and standard deviation) for the SPAT’s 13 item total scores. Structural validity was examined with exploratory factor analysis, and internal consistency with Cronbach’s alpha coefficients. The mean SPAT total scores across different SPs and scenarios were compared using one way analysis of variance (ANOVA). Convergent validity was determined by correlating SPAT with the post-clinical clerkship obstructive structured clinical examination (post-CC OSCE) total scores using Pearson’s correlation coefficient.

**Results:**

Of the 831 assessment sheets, 36 with missing values were excluded, leaving 795 for analysis. Thirty-five SPs, excluding one SP who quit in 2014, completed 795 assessments, for a response rate of 95.6%. Exploratory factor analysis revealed two factors, communication and physician performance. The overall Cronbach’s alpha coefficient was 0.929. Significant differences in SPAT total scores were observed across SPs and scenarios via one-way ANOVA. A moderate correlation (*r* =.212, *p* <.05) was found between SPAT and post-CC OSCE total scores, indicating convergent validity.

**Conclusions:**

Evidence for the validity of SPAT was examined. These findings may be useful in the standardization of SP assessment of the scenario-based clinical performance of medical students.

**Supplementary Information:**

The online version contains supplementary material available at 10.1186/s12909-024-05386-2.

## Background

Medical students need to acquire clinical skills as an essential component of clinical practice, supervised by residents and attending physicians, and clinical performance assessment is an increasingly prevalent component of medical education [1]. Simulated and standardized patient-based performance assessments, including both technical and non-technical skills, are commonly used to keep patients safe while providing students with effective learning experiences [2, 3]. The use of simulated patients (SPs) in medical education dates back to the 1960s, when neurologist Howard Burroughs introduced the concept as a means of assessing clinical performance [4]. An SP is a person who has been trained to accurately represent a patient and present consistent verbal and nonverbal communication, personality traits, emotions, and physical findings [4]. The use of SPs in medical education emphasizes patient safety and provides students with opportunities for immersion and interaction in patient care scenarios that closely resemble real clinical practice [5]. The training of SPs is critical to the quality and accountability of medical education and transition to clinical practice [4]. A key advantage of SPs is the feedback they provide to students from the perspective of the patient. Feedback in medical education is defined as “specific information about the comparison between a trainee’s observed performance and a standard, given with the intent to improve the trainee’s performance“ [6, 7]. In particular, feedback from SPs is rated with the same or greater positivity than feedback from physicians [8, 9]. Feedback from SPs is valuable as it encompasses both non-verbal behaviors, such as open body posture and appropriate facial expressions, and verbal interactions, including the use of open and closed questions, encouragement of questions, and requests for clarification [6, 10, 11]. Studies have shown that medical students can improve both non-verbal and verbal communication through SP interactions, enhancing their ability to engage effectively with patients [12–14]. Moreover, the unique perspective of SPs enables a focus on patient-centered feedback, which some students may find more constructive, even when it is negative, compared to feedback from physicians [15]. Implicit feedback during student actions can also facilitate feedback acceptance and promote professional identity formation, underscoring the multifaceted impact of SP feedback on medical education [16]. The incorporation of SPs into medical education not only emphasizes patient safety but also offers students immersive and interactive patient care scenarios that closely resemble real clinical practice [17, 18]. The training of SPs is critical to the quality and accountability of medical education and the transition to clinical practice [19]. This makes the assessment of medical students by SPs increasingly meaningful. Nevertheless, few reports have described the validity of the assessment tools used by SPs [20, 21].

In Japan, the participation of SPs in assessing medical student performance is playing an increasingly important role in medical education in Japan [11], and assessment by SPs is becoming indispensable as a patient-centered evaluation [22]. To date, however, few reports have investigated the development of assessment tools, including testing of the validity and reliability of the clinical performance of medical students using SPs [23].

With the growing importance of SPs in assessing medical student performance, we aimed to develop a validated SP assessment tool (SPAT) for the clinical performance of medical students. This initiative seeks to address the gap in validated assessment tools involving SPs, contributing to more standardized and reliable assessments of clinical performance.

## Methods

### Design

The study was conducted under a longitudinal survey design.

### Setting

The study investigated the use of SPs in medical education at Keio University School of Medicine in Tokyo, Japan. In Japan, students are required to complete a six-year medical school education after three years of senior high school. The medical school curriculum begins in April and is regulated. Students take basic education courses in the first year, followed by specialized education courses from the second to sixth year. For example, in the second year, students study anatomy and physiology, followed by basic and social medicine such as pathology and public health in the third year, and clinical medicine such as internal medicine in the third and fourth years. Before beginning clinical practice, students in the second half of the fourth year take a CBT (computer-based test) and the OSCE (objective structured clinical examination), and if they pass are certified as student physicians. The post-CC OSCE (post clinical clerkship objective structured clinical examination) has been in place since 2020, and CBT and OSCE will become official starting in 2023.

The program started in 2013, and over the course of 8 years involved 36 participating SPs who interacted with 831 medical students during their clinical training. The mean length of SP encounters among students was 6.5 min, and each consisted of one medical interview based on any of 23 scenarios of common diseases (Table 1). These consist of common diseases, excluding shock and cardiac arrest, from the 37 symptoms described in the Japanese Model Core Curriculum for Medical Education [24], followed by self-feedback by the student, as well as peer-reviewed feedback from other students and from the SP lasting 4–5 min per student. In this study, although no specific feedback tool was employed, the approach to feedback was informed by literature that emphasizes the importance of focusing on biomedical aspects as well as maintaining a balance between positive and negative feedback [25]. As the amount of time for this oral feedback was limited, SPAT was used as supplement to it. SPs were instructed to provide oral feedback focusing on 2–3 behavior-level aspects, making it concrete, interpretable, and actionable for students [26]. This approach was designed to complement the SPAT by offering immediate, personalized feedback to enhance learning outcomes. A physician faculty member facilitated the process of self-feedback and peer feedback among students to foster a constructive feedback environment. He summarized the students’ clinical performance and gave a brief lecture on each scenario.

**Table 1:** 21 Scenarios in the analysis

Pleuritis	Infective endocarditis
Irritable bowel syndrome	Wolff-Parkinson-White syndrome
Benign prostatic hyperplasia	Vasovagal reflex
Diabetes mellitus	Transient ischemic attack
Chronic renal failure	Arteriosclerosis obliterans
Pulmonary hypertension	Pulmonary thromboembolism
Variant angina pectoris	Subarachnoid hemorrhage
Cholelithiasis	Anisakiasis
Iron deficiency anemia	Benign paroxysmal positional vertigo
Acute pyelonephritis	Acute cholangitis
Hyperthyroidism	
Example scenarios He had been experiencing intermittent epigastric pain (pain in the groin) for several days, which abated spontaneously. Today, the patient came to our hospital because his had been treated for angina pectoris 5 years ago, and sublingual nitroglycerin had not relieved her symptoms. He began to have pain in the area of his solar plexus, felt chilly, and then suddenly became feverish. At the time of consultation, his had a fever of 38.8 degrees Celsius. He had undergone coronary angioplasty (catheterization to widen a narrowed coronary artery with a balloon) for exertional angina pectoris 5 years ago. Medications: aspirin, Plavix, Norvasc, Renivace, Lipitor. Life history: no smoking, no alcohol consumption, nothing else of note. Since the patient has a history of angina pectoris in the past, it is naturally tempting to consider a recurrent attack of angina pectoris, but the key point is whether other diseases can be considered there as well. In particular, since there is no fever with angina pectoris, can we consider infection? The first complaint, “What happened to you today?” In response to the first complaint…” “I have a pain in my solar plexus.” If you are asked to elaborate a little more… (1) I had a pain in the area of my solar plexus several times a day for a few days, but it had gotten better without any treatment. I have had catheter treatment for angina in the past, so I thought it might be angina again and tried sublingual nitroglycerin, but it didn’t work at all.	

The gout/hyperuricemia and lung cancer scenarios were excluded from the scenarios in the analysis because they were used only four and two times, respectively

### Assessment forms and data collection

In 2008, an assessment form utilizing a 5-point Likert scale consisting of 28 items was introduced (Supplemental file 1). This formulation was developed based on medical interview protocols [27]. However, the extensive number of 28 items proved cumbersome for SPs and led to increased variability in assessments. In 2013, SPAT was streamlined to 13-item version, and in 2014, shifted to a 6-point scale to refine feedback specificity (Table 1). This form, used in this study, assesses performance on a 6-point scale, with scores reflecting levels from physician-standard to inappropriate for medical students. The rating scale was defined as follows: a score of 6 indicates performance at the level of a physician; 5 signifies excellence as a medical student; 4 denotes acceptable performance as a medical student; 3 suggests some issues as a medical student, though not critical; 2 indicates performance that is inappropriate as a medical student but improvable; 1 is deemed inappropriate for a medical student. Responses were provided through a general assessment from the patient’s perspective. For compound statement items like Item 1, “Greeted, introduced self, confirmed the patient’s full name, date of birth, and age,” SPs were instructed on a graduated scoring approach. For each behavior not performed, a point would be deducted, resulting in a score that reflects the number of behaviors successfully completed. In contrast, for items with binary responses, such as “Established good eye contact,” SPs were trained to utilize the 6-point Likert scale rather than a simple yes/no dichotomy. This scale enables SPs to assess the quality of eye contact in a more graded manner, considering aspects such as consistency and appropriateness of eye contact, rather than a binary presence or absence. This approach ensured nuanced students’ performance assessments. To ensure consistency, SPs were trained to enact multiple scenarios throughout 2013. New SPs who joined mid-study underwent extensive practice and calibration with existing SPs to familiarize themselves with the assessment form and ensure uniformity in assessments. SPAT was filled out after oral feedback was given to the students from the SP’s point of view.

### Data analysis

To evaluate the validity of SPAT, the authors conducted various tests and analyses, including a pilot study for content validity, descriptive statistics for the scores, exploratory factor analysis for construct validity, internal consistency using Cronbach’s alpha, and comparison of mean total scores across SPs and scenarios using one-way analysis of variance (ANOVA). Relationships between scores on the SPAT in 2021 and the post-CC OSCE in 2022 were also evaluated using Pearson’s correlation coefficients. The standardized patient assessment form used in the post-CC OSCE in Japan consists of five items that are rated on three levels, namely 2-good, 1-neutral, and 0-bad. This type of assessment was given for a combination of the five aforementioned items and a global rating of the medical student’s clinical performance on a 6-point scale, ranging from 6-very good, 5-good, 4-normal, 3-slightly bad, 2-bad, and 1-very bad. The five assessment items of the post-CC OSCE, which are confidential, encompass elements of both nonverbal and verbal communication. While there is partial overlap between the items of the SPAT and those of the post-CC OSCE, the SPAT features more detailed and specific questions. The post-CC OSCE took 16 min, consisting of 12 min dedicated to a medical interview and physical examination and 4 min for a presentation to the supervising physician. The standardized patient was responsible for assessing the medical student’s performance in the first 12 min of the post-CC OSCE.

The study used IBM SPSS Statistics version 27 and a significance level of 95% for all data analysis. This study was approved by the Keio University Research Ethics Committee (No. 20,211,156), and was performed in accordance with the Declaration of Helsinki. All participants were given the opportunity to opt out in the web-page of the medical education center at Keio University. Informed consent was obtained from all participants.

## Result

This study analyzed the total score of SPAT used in the SP encounter program and post-CC OSCE in Japan. Two further scenarios were rarely used in the program, leaving 21 scenarios for inclusion in the analysis (Table 1). We excluded one SP who involved in the aforementioned scenario and quit in 2014. Finally, 35 SPs participated, consisting of 19 women and 16 men, ranging in age from 35 to 93 years, with 1–15 years of experience as SPs (Table 2). After exclusion of two scenarios and sheets with missing values, 795 of the 831 assessment sheets collected were analyzed, with a valid response rate of 95.6%.

**Table 2:** Participant SPs characteristics

Age* in years	n (%)
Average (range)	66.1 (35–93)
30–39	2 (5.7)
40–49	2 (5.7)
50–59	9 (25.7)
60–69	5 (14.3)
70–79	12 (34.3)
80–89	4 (11.4)
90–99	1 (2.9)
**Gender**	
Male	16 (45.7)
Female	19 (54.3)
**Years of experience**	
1–5 years	14 (40.0)
6–10 years	8 (22.9)
11–15 years	13 (37.1)

* Age at the time of quitting being an SP (simulated patient)

A 13-item SPAT was designed to assess different aspects of each medical student’s performance during a SP encounter. Item 1 focused on the introduction to the medical history interview and how well the student greeted and introduced themselves to the patient. Item 2 evaluated the student’s cleanliness and appearance, which can impact the patient’s trust in the student as a healthcare provider. Items 3–8 were designed to assess basic communication skills with patients, such as the student’s ability to establish rapport, listen actively, and respond appropriately to the patient’s needs and concerns. These items are important as effective communication is a key component of quality healthcare delivery. Items 9–13 focused on the student’s ability to gather medical information from the patient (Table 3). This included assessment of the student’s ability to ask appropriate questions, elicit a thorough history, and obtain relevant physical examination findings. The items were designed to assess how well the student was able to gather the information necessary to make an accurate diagnosis and develop an appropriate treatment plan.

**Table 3:** Statistical analysis

	Items	Mean	SD	Factor 1	Factor 2
1	Greeted, introduced self, confirmed the patient’s full name, date of birth, and age	5.37	0.71	0.718	
2	Wore a clean white lab coat, gave off a sense of cleanliness such as in hairstyle, clothes and make up	5.33	0.70	0.732	
3	Responded to the patient’s remarks and kept a professional distance and attitude	5.12	0.75	0.859	
4	Spoke at just the right volume	5.04	0.86	0.862	
5	Established good eye contact	5.11	0.84	0.761	
6	Built a rapport with the patient	4.91	0.81	0.585	
7	Was empathetic towards the patient throughout the whole encounter	4.61	0.92	0.465	
8	Listened without interrupting and asked if I have any additional questions or concerns	4.47	0.89		0.573
9	Actively listened to the history of present illness	4.51	0.90		0.684
10	Avoided use of medical jargon and intelligibly explained	4.95	0.72		0.416
11	Gave a diagnosis properly	4.31	1.11		0.979
12	Counseled me appropriately and told me what happens next about examinations or treatments	4.45	0.90		0.893
13	I would like to visit this doctor again	4.59	0.89		0.720
	Cronbach’s alpha	0.929		0.897	0.897

Means and standard deviations for all 13 items are shown in Table 3; a ceiling effect was observed for 2 of the 13 items, but was not excluded because SPs are an essential item in assessing medical students.

In exploratory factor analysis (EFA), the KMO value was calculated as 0.935, and Bartlett’s sphericity test for sphericity was significant (χ2 = 6832.13, df = 78, *p* <.001). The.

EFA revealed two factors: “communication” and “physician performance”. These two factors indicated a cumulative contribution rate of 60.47%, with individual contribution rates of 52.1% for factor 1 and 5.73% for factor 2. The “communication” factor consisted of 7 items related to communication skills, including physical distance between the SP and the medical student, and listening attitude, while the “physician performance” factor consisted of 6 items related to the medical student’s performance as a physician. The overall Cronbach’s alpha coefficient was 0.929, with factor 1 at 0.897 and factor 2 at 0.897, indicating good internal consistency of the tool (Table 3). A one-way ANOVA using the total score of SPAT showed a significant difference in mean total score among SPs (F(34, 760) = 16.79, *p* <.001 ) and among scenarios (F(20, 774) = 11.39, *p* <.001) (Table 4). Feedback from the study showed that the items in the SPAT were easy to understand. The correlation coefficient between the total score in the program with SPs in 2021 and the post-CC OSCE with standardized patients in 2022 was 0.212 (*p* <.05), indicating a moderate relationship between the two scores.

**Table 4:** One way ANOVA for the difference in mean total scores of the SPAT by SP and scenario

Source	Sum of squares	df	Mean square	F	*p*
SPs					
Between groups	22468.92	34	660.85	16.79	< 0.001
Within groups	29919.22	760	39.37		
Total	52388.14	794			
**Scenarios**					
Between groups	11910.05	20	595.51	11.39	< 0.001
Within groups	40478.10	774	52.30		
Total	52388.15	794			

SPAT: Simulated patient assessment tool; SP: Simulated patient; ANOVA: Analysis of variance

## Discussion

This study provides evidence for the validity of the SPAT as a tool for assessing the performance of medical students in a SP encounter program. The development of the SPAT, which includes a two-factor structure of communication and physician performance and high internal consistency, increases the validity and reliability of the assessment. Additionally, the findings show that the scores of medical students vary depending on the scenario and SP, indicating the need for standardization. This can be done by informing SPs in advance about the assessment and by considering the scenario used in high-stakes examinations.

SP assessment typically focuses on non-medical aspects [28, 29]. The development of the SPAT in this study offers a more comprehensive and valid way to assess the clinical performance of medical students. It achieves this by incorporating both communication skills and physician performance. SP assessments are often in a checklist format, which is reported to be a time- and cost-effective way of assessing physician communication skills [28]. In a review of medical communication measures by Schirmer et al. [30], SP ratings were shown to have been developed to capture specific behaviors such as communication, satisfaction with the session, trust in patient-physician communication, and counseling [31–34]. Additionally, negative communication was rated as indicating less competency as a physician [35]. The use of a global rating instead of a checklist provides more in-depth feedback to medical students, allowing them to see how their communication and physician performance is perceived from a patient’s perspective. Thus, our findings confirm the validity and reliability of the SPAT, including the global rating provided by Likert scale items for performance as a physician as well as communication skills.

The collaborative effort between SPs and educators in developing the SPAT, which involved reducing the item count from 28 to 13, enhances its practicality [36]. Moreover, demonstrating the robustness of the constructs and their correlation with official test assessments could have further solidified the instrument’s validity. Highlighting the global relevance of patient involvement in physician training, this study extends the application of our findings beyond Japan, aiming to contribute to the broader discourse on enhancing clinical education through validated assessment methodologies.

We particularly emphasize the significance of items 3–7, which assess key interpersonal skills essential for building relationships and sharing information with patients. These skills directly contribute to patient-centered care, which has been shown to positively impact patient satisfaction and outcomes, as highlighted in “The impact of patient-centered care on outcomes” [31]. Items 9–13 assess skills vital for building trust with patients, an aspect directly linked to patient satisfaction with their perception of a knowledgeable and trustworthy physician [37–39]. Considering that trust is cultivated through a unique interaction of medical expertise and humaneness, the two-dimensional structure of communication skills and physician performance as a foundation for the physician-patient trust relationship is internationally validated as an appropriate measure. These potential influences of oral feedback from SPs on student learning outcomes and SPAT results was not examined in the current study. This confirmation will in turn help medical students improve their skills and potentially lead to a reduction in medical errors in the future. The use of a SP as the assessor provides unique and meaningful perspectives, as patients who rate their physicians higher in competence have been shown to make fewer malpractice claims against them [40].

This finding highlights the importance of standardizing the SP assessment process, and suggests that the performance ratings of SPs may vary depending on the scenario and SP encounter. To address this issue, we recommend informing standardized patients in advance about the evaluation, as well as providing feedback on the leniency or severity of their ratings which may help in identifying appropriate scenarios for high-stakes exams such as national examinations [41, 42]. This can improve the standardization of the SP assessment process and ensure that medical students are assessed fairly and accurately.

In addition, it is important to train SPs to rate consistently and to avoid any bias that may be related to gender, age, or other demographic factors. For example, a U.S. study reported that third- and fourth-year female medical students rated significantly higher than male medical students on rating of empathy demonstrated during OSCE by SPs, regardless of gender or ethnicity [43]. In a longitudinal study in Germany, SPs rated female medical students higher than male medical students on all aspects tested during the OSCE, including empathy, content structure, verbal expression, and nonverbal expression [44]. Another study reported that older SPs, regardless of gender, are more likely to assign lower scores to medical students than younger SPs on all survey questions [23]. By considering these factors, medical schools can develop standardized assessment through regular training and assessment of SPs, as well as by using standardized scenarios that are designed to reduce potential bias in the assessment process, and provide more accurate feedback to medical students on their communication and performance skills. Our development of the SPAT globally seeks to mitigate potential biases associated with SP characteristics and scenarios, standardizing evaluations to improve the reliability and utility of SP feedback within the framework of medical education.

### Limitations

This study has several limitations. First, the external validity of the study should be considered since it was conducted in a single sample of medical schools. However, given the gender distribution of medical students in Japan, this is not a highly skewed attribute [45]. Additionally, the fact that all SPs responded 100% non-anonymously in both class and post- CC OSCE reduces the risk of response bias [46]. The results of the post-CC OSCE are also not highly skewed given the distribution of Japanese medical students. Second, the correlation between the score of the program with a SP, which was conducted as a formative assessment, and the post-CC OSCE, which was conducted as a summative assessment, was only moderate. Some medical students may have experienced growth or conversely become indolent between the first and second years of the study, which could have weakened the correlation. Additionally, the post-CC OSCE is an exam that is influenced by time constraints, which may have affected medical student performance [47]. Considering these limitations, a key strength lies in the methodological rigor with which the SPAT was developed and validated. The collaborative process between SPs and educators not only ensured the tool’s practicality but also its alignment with the nuanced requirements of clinical education. Furthermore, the study’s design, involving a diverse range of SP encounters and comprehensive feedback mechanisms for 8 years, offers a robust framework for assessing and enhancing medical students’ clinical skills. Despite being conducted within a single medical school, the meticulous attention to the representation of gender distribution and the avoidance of response bias through non-anonymous SP feedback add layers of reliability and validity to the findings. Moreover, the moderate correlation observed between the SPAT scores and the post-CC OSCE results, while highlighting a limitation, also underscores the complexity of measuring clinical performance. This aspect of the study illuminates the multifaceted nature of clinical skills development, emphasizing the importance of formative assessments like SPAT in identifying areas for improvement and guiding student learning.

SPAT can help medical students improve their clinical performance when it is used in medical student classes. We also believe that the results can be fully utilized in standardizing assessment of medical students’ performance with SPs and in providing feedback to medical students from a patient-centered perspective that is independent of SPs or scenarios.

## Conclusion

We developed a validated simulated patient assessment tool (SPAT) to assess and provide evidence for medical students’ clinical performance. The significant differences in scores between raters and scenarios may provide new insights into the standardization of SPs and the selection of scenarios for high-stakes testing. These insights contribute to the ongoing efforts to improve the reliability and validity of clinical skills assessment in medical education, emphasizing the importance of rigorous tool development and validation processes.

### Electronic supplementary material

Below is the link to the electronic supplementary material.

Supplementary Material 1

## Data Availability

The data that support the findings of this study are available from the corresponding author [JH], upon reasonable request.
